# Perception of adults toward electronic cigarettes: a cross-sectional study from Jordan

**DOI:** 10.1017/S1463423621000062

**Published:** 2021-01-28

**Authors:** Muna Barakat, Areej M. Assaf, Raja’a Al-Qudah, Samar Thiab, Manar Alhamed, Hala J. Al-Obaidi, Feras J. Jirjees, Iman Basheti

**Affiliations:** 1 Department of Clinical Pharmacy and Therapeutics, Faculty of Pharmacy, Applied Science Private University, Amman, Jordan; 2 Department of Biopharmaceutics and Clinical Pharmacy, Faculty of Pharmacy, The University of Jordan, Amman, Jordan; 3 Clinical and Practice Research Group, School of Pharmacy, Queen’s University Belfast, Belfast, UK; 4 Department of Pharmacy Practice and Pharmacotherapeutics, College of Pharmacy, University of Sharjah, Sharjah, United Arab Emirates

**Keywords:** beliefs, Jordan, E-cigarettes, E-cig, knowledge, smoking

## Abstract

**Introduction:**

The rate of Jordanian tobacco smokers has been reported to be one of the highest rates in the world. The electronic cigarette (E-cig) has become an option, or an alternative, to tobacco cigarette smoking. This study was aimed to measure the perception of Jordanian adults toward E-cig use.

**Methods:**

A cross-sectional study design was used. A self-administered survey was developed and validated to solicit anonymous responses from the study participants. A convenience sample (*n* = 984) was recruited electronically through social media platforms. Descriptive statistics and correlation analyses were completed using the Statistical Package for the Social Sciences (SPSS).

**Results:**

More than half of the participants (53%) were females, and almost all participants had heard about E-cig (99.2%), mainly from their friends (40%) and social media (34.5%). About half of the participants were nonsmokers and around one-third of them (33.1%) were current E-cig users. The majority of the participating E-cig users had replaced tobacco with E-cig (56.4%)/All the E-cig users reported positive beliefs toward the E-cig as a safer alternative for tobacco smoking. About 45% of participants believed that the E-cig is helpful in tobacco smoking cessation, but should be highly regulated.

**Conclusion:**

This study illustrated a significant prevalence of E-cig usage among Jordanian adults. E-cig users perceived E-cig as a safer and cheaper alternative to tobacco smoking and that it helps in tobacco smoking cessation. However, health awareness campaigns are needed for the entire Jordanian community about E-cig use, related emerging health findings, and how to promote tobacco smoking cessation.

## Introduction

In 2015, the World Health Organization (WHO) reported that over 1.1 billion people smoked tobacco, with males using tobacco products more than females (WHO, 2015). The prevalence of tobacco smoking appears to be increasing in the Eastern Mediterranean Region and the African Region (WHO, 2015). In Jordan, the percentage of tobacco smokers is one of the highest in the world, reaching 70.2% among males (WHO, 2015). Looking at these percentages, Jordan adopted the National Tobacco Control Strategy for 2017–2019, which is based on the implementation of a comprehensive set of tobacco control measures approved by the WHO (Marquez *et al*., 2019). The strategy aims to diminish tobacco usage by 30% by 2025 (Marquez *et al*., 2019).

The use of electronic cigarettes (E-cig) has become an alternative option for tobacco smoking (NIDA, 2019). According to the National Cancer Institute and the National Institute on Drug Abuse, E-cig is defined as a battery-operated device that people use to inhale an aerosol (e-liquid), which typically contains nicotine (though not always), flavorings, and other chemicals (NIDA, 2019; National Cancer Institute, 2019). E-cig has been invented as a tobacco smoking cessation tool, due to the common belief in its safety compared to tobacco smoking (Grana *et al*., 2014). Some studies suggested that E-cig might be less harmful than tobacco smoking, especially when people who regularly smoked tobacco switch to them as a therapeutic replacement (NIDA, 2019).

However, nicotine in any form is a highly addictive drug (NIDA, 2019). Levine *et al*. reported that E-cig can even prime the brain’s reward system, putting vapers (E-cig users) at risk for addiction to other drugs (Levine *et al*., 2011). In addition, E-cig use exposes the lungs to a variety of chemicals, including added flavors to e-liquids, volatile organic compounds, and some heavy metals such as nickel, tin, and lead, which are produced during the heating/vaporizing process (Sleiman *et al*., 2016). Those chemicals have been found to induce inflammatory responses similar to those induced by tobacco smoking (Sundar *et al*., 2016; Kaur *et al*., 2018). Based on recent evidence, exposure to the constituents of E-cig aerosols might result in serious respiratory injuries and complications including asthma, chronic obstructive pulmonary disease (COPD), and severe inflammation of the lungs (Kaur *et al*., 2018; Chatterjee *et al*., 2019; CDC, 2020a).

Many research studies have been conducted to evaluate the beliefs and attitudes of people toward the E-cig (Singh *et al*., 2017; Buczek *et al*., 2018; Hart *et al*., 2018; Tamimi, 2018; Dwedar *et al*., August 2019; Wang *et al*., 2019). In Egypt, for example, most of the healthcare providers believed that the E-cig is unsafe and does not help in tobacco smoking cessation (Dwedar *et al*., August 2019). Besides, more than half of the healthcare providers, and the general population, agreed that using E-cig is a public health concern (Dwedar *et al*., August 2019). In South East England, a study aimed to explore how E-cig is perceived by a group of E-cig users found that E-cig was used as a therapeutic aid to stop or cut down on tobacco smoking (Tamimi, 2018). Some users adopted the E-cig as a hobby and a social activity (Tamimi, 2018). Additionally, it was found that most E-cig users opted to use E-cig to improve their health (Tamimi, 2018). In China, about 88% of the study subjects were found aware of E-cig, especially the current tobacco smokers, and ex-smokers who had a higher level of awareness and use of E-cig than others (Wang *et al*., 2019).

It is worth mentioning that the majority of E-cig users perceived their health to be in a better state since they began to use it (i.e. vaping process) (Hart *et al*., 2018). Currently, limited published data addressing E-cig perceptions and usage among Jordanians, a population titled to become number one when it comes to smoking rates, calls for an urgent investigation. Therefore, this study was aimed to measure the prevalence of E-cig usage among Jordanian adults, and to assess their knowledge, beliefs, and practice toward E-cig use.

## Materials and methods

### Study design and participants

A cross-sectional study was conducted between October and December 2019. A self-administered electronic questionnaire was developed and validated (face and content validation) to solicit anonymous responses from the study participants. Eligible participants included Jordanian adults interested in participation. Participants were recruited online through social media platforms: Facebook, Twitter, LinkedIn, and WhatsApp. Participants who were unable to open the questionnaire (due to technical issue) and asked to send them the questionnaire were contacted via their provided institutional emails. The used invitation statement for sample recruitment was: “*We are a group of keen researchers from different Jordanian universities would like to invite you to fill this questionnaire, which aims to measure the prevalence of electronic cigarette use among Jordanian adults, and to assess their knowledge, beliefs and practice towards electronic cigarette. The questionnaire consists of three sections only, which will require 5–7 minutes of your time to answer, knowing that the questionnaire does not require writing the name or any other private information. The information will be treated in strict confidence and will be used for the purposes of scientific research.*”

A comprehensive description of the study was introduced on the first page of the questionnaire, mentioning that the participation in the questionnaire is voluntary and their response would be treated confidentially. Potential participants who completed the survey were considered to have given informed consent for study participation.

### Questionnaire development

The questionnaire was developed after reviewing validated surveys found in the literature and designed using the general principles of good survey design (Boynton and Greenhalgh, 2004; Brown *et al*., 2014; Couraud *et al*., 2018). This online questionnaire was created using the technology of Google Forms provided by Google*™.* The questionnaire contained multiple-choice questions that can be completed within 15 minutes, and it was administered in Arabic being the official language of the Jordanian population. This questionnaire contained three parts: the first part (Part A) was designed to collect data regarding participants’ sociodemographic characteristics; Part B was designed to evaluate the knowledge and beliefs about E-cig, and Part C was prepared to assess the practice and attitude of Jordanian adults toward E-cig. The questionnaire is available from the corresponding author upon request.

### Validation and reliability

The initial draft of the questionnaire was evaluated by the research team members and amended to enhance the clarity and readability of the study questions. The evaluation of validity and reliability of the questionnaire was conducted by a professional committee of clinical pharmacists and a statistician, confirming the applicability for the Jordanian population. The questionnaire was translated from English to Arabic and back-translated by two senior academic staff members who were ﬂuent in both languages. The questions were free from medical jargons or hard terminology. Then, the questionnaire was evaluated and validated via a pilot sample of 25 academics and 25 nonacademic participants during a month pilot study to ensure comprehension, clarity, readability, and acceptability of the survey. Accordingly, modifications to the questions were made as needed before its implementation. The Cronbach’s alpha coefficient was tested for the study instrument (equals to 0.88), which indicates the internal consistency and reliability.

### Measures

#### Sociodemographic

The sociodemographic information including age, gender, educational level, and work status was requested. Also, in the case participants were tobacco smokers, they were asked about their tobacco smoking history and the heaviness of their smoking (the average number of tobacco cigarettes smoked per day).

#### Beliefs toward the E-cig

Participants were asked if they agreed or disagreed with certain questions related to their beliefs and attitudes toward the E-cig. Questions focused on their beliefs toward the safety of the direct or passive (indirect exposure to the E-cig vape) vaping E-cig compared to tobacco smoking. Participants were asked if they believed that the E-cig was a helpful aid for tobacco smoking cessation and if they switched from tobacco smoking to the E-cig before. More questions were asked related to the impact of the E-cig on the participants’ general health, public health, and its addictive potential. Finally, this section included questions about participants’ beliefs toward the E-cig cost and cost-effectiveness, uses in public areas, and their selling regulations. The options followed a Likert scale including “strongly agree”, “agree”, “neutral”, “disagree”, “strongly disagree”. As some participants may not have the needed knowledge about the E-cig or the fact that it presents a health issue, the option neutral was added as an option to the Likert scale, providing the participants with the needed response while answering such questions.

#### Attitudes and knowledge toward E-cig

The participants were asked about their knowledge and attitude toward the E-cig by specific questions. The questions were about how they heard about the E-cig for the first time, if they ever tried using it before and if they were current solo or tobacco combined users. Accordingly, participants were classified to be as “never used the E-cig” and the “E-cig user who ceased its use” and lastly the “the current E-cig user”.

### Sample size

The most recent demographic statistics belonging to citizens living in Jordan showed that 10.554 million people live in the country (Department of Statistics). Based on that, the sample size was calculated using a margin of error of 5%, confidence level of 95%, and response distribution of 50%, giving a minimum sample size of 385 (Taherdoost, 2017). It was decided to increase the number to around 995 to take into account missing responses and other unknown issues that might arise.

### Statistical analyses

Statistical analyses were conducted using the Statistical Package for the Social Sciences (SPSS) Version 24.0 for Windows (SPSS, Inc., Chicago, IL, USA). Descriptive statistics including percentages; means and frequency distribution were calculated for each of the questions. Categorical variables, expressed as a proportion (%), were analyzed using the Chi-square test. Descriptive and univariate correlation analyses with the Pearson correlation coefficient (r) was used for the correlation at the 5% significance level. A *P*-value of <0.05 represented a significant difference. Simple and multivariant linear logistic regression was used to explore significant correlations between the E-cig use and different variables.

## Results

### Sociodemographic characteristics

Out of the total 995 completed questionnaires, 11 forms (1.1%) were excluded from the study due to being incompletely answered, accordingly, 984 (98.9%) of the answered questionnaires were included in the study analysis. Table 1 showed the demographic characteristics of the study participants. Among the total study sample, more than half (53%) of the participants were females. The participants had a wide age range, almost one-third (*n* = 367, 37.2%) of the participants were aged 26–35 years and 26.3% were aged 36–50 years. The majority of participants had a university degree, either bachelor’s or postgraduates (63.9% and 16.2%, respectively). More than half of the participants were employed/self-employed (*n* = 535, 54.3%), and many (*n* = 211, 21.4%) were students. About 50% (*n* = 517) were married, and most of the participants lived in the capital of Jordan, Amman (*n* = 811, 82.3%).

**Table 1.** Demographic characteristics of the study participants (*n* = 984)

Characteristic	n (%)						
	Nonsmokers (*n* = 492)	Tobacco smokers alone (*n* = 166)	E-cigarette users (*n* = 326)			Total *n* = 984	*P*-value*
			Replaced tobacco with E-cig (*n* = 184)	Dual users for E-cig+ tobacco (*n* = 52)	E-cig users without tobacco smoking history (*n* = 90)		
**Gender**							
• Female	378 (38.4)	74 (7.5)	18 (1.8)	16 (1.6)	37 (3.8)	523 (53.1)	
• Male	114 (11.6)	92 (9.4)	166 (16.9)	36 (3.7)	53 (5.4)	461 (46.8)	<0.001
**Age**							
• 18–20 years	43 (4.4)	11 (1.12)	10 (1.0)	4 (0.4)	17 (1.7)	85 (8.6)	
• 21–25 years	110 (11.2)	24 (2.4)	20 (2.0)	7 (0.7)	23 (2.3)	184 (18.7)	<0.001
• 26–35 years	146 (14.8)	56 (5.7)	96 (9.8)	27 (2.7)	42 (4.3)	367 (37.3)	
• 36–50 years	136 (13.8)	54 (5.5)	51 (5.2)	10 (1.0)	8 (0.8)	259 (26.3)	
• 51–65 years	53 (5.4)	19 (1.9)	6 (0.6)	3 (0.3)	0 (0.0)	81 (8.2)	
• <65	4 (0.4)	2 (0.2)	1 (0.1)	1 (0.1)	0 (0.0)	8 (0.8)	
**Educational level**							
• No schooling completed	10 (1.0)	0 (0.0)	4 (0.4)	2 (0.2)	0 (0.0)	16 (1.6)	<0.001
• Secondary school	53 (5.4)	14 (1.4)	16 (1.6)	7 (0.7)	25 (2.5)	115 (11.7)	
• Diploma	25 (2.5)	6 (0.6)	12 (1.2)	6 (0.6)	9 (0.9)	58 (5.9)	
• Vocational education	3 (0.3)	3 (0.3)	1 (0.1)	0 (0.0)	0 (0.0)	7 (0.7)	
• Bachelor’s degree	317 (32.2)	111 (11.3)	114 (11.6)	32 (3.3)	56 (5.7)	630 (64.0)	
• Postgraduate degree	84 (8.5)	32 (3.3)	37 (3.8)	5 (0.5)	0 (0.0)	158 (16.1)	
**Work status**							
• Employed/Self-employed	209 (21.2)	97 (9.9)	147 (14.9)	35 (3.6)	47 (4.8)	535 (54.4)	
• A homemaker	96 (9.8)	23 (2.3)	6 (0.6)	4 (0.4)	1 (0.1)	130 (13.2)	<0.001
• A student	120 (12.2)	30 (3.1)	18 (1.8)	6 (0.6)	37 (3.8)	211 (21.4)	
• Retired	21 (2.1)	4 (0.4)	3 (0.3)	3 (0.3)	0 (0.0)	31 (3.2)	
• Others	46 (4.7)	12 (1.2)	10 (1.0)	4 (0.4)	5 (0.5)	77 (7.8)	
**Marital status**							
• Single	225 (22.9)	66 (6.7)	70 (7.1)	24 (2.4)	51 (5.2)	436 (44.3)	
• Married	252 (25.6)	95 (9.7)	109 (11.1)	26 (2.6)	35 (3.6)	517 (52.5)	
• Divorced/Widowed	15 (1.5)	5 (0.5)	5 (0.5)	2 (0.2)	4 (0.4)	31 (3.2)	0.018
**Current place of residence**							
• Amman	420 (42.7)	135 (13.7)	147 (14.9)	39 (4.0)	70 (7.1)	811 (82.4)	
• Central Jordan area	41 (4.2)	13 (1.3)	24 (2.4)	8 (0.8)	12 (1.2)	98 (10.0)	0.125
• Northern Jordan area	26 (2.6)	16 (1.6)	12 (1.2)	4 (0.4)	8 (0.8)	66 (6.7)	
• Southern Jordan area	5 (0.5)	2 (0.2)	1 (0.1)	1 (0.1)	0 (0.0)	9 (0.9)	

* Chi-square test (*P*-value<0.05).

As demonstrated, the study participants were stratified for different categories (nonsmokers 50%, tobacco smokers 16.9%, E-cig users 33.1%). Out of the 326 participating E-cig users, half (56.4%) of them had replaced tobacco completely with E-cig (*n* = 184), while the rest were either dual users for E-cig and tobacco (*n* = 52) or E-cig users without a tobacco smoking history (*n* = 90). Statistically, there was a significant difference between the subgroups over most demographic characteristic variables (*P*-value <0.05). Among the E-cig users and tobacco smokers’ subgroups, most of them were male aged between 26 and 35 years and had a bachelor’s degree. Most of them were married, live in Amman, and were employed. All the participating E-cig users were vaping nicotine-containing e-liquids. On the other hand, tobacco smokers were varied in the heaviness of their smoking; 21.1% of them were heavy smokers (smoking >20 cigarettes/day), while 34.3% reported being moderate (smoking 10–20 cigarettes/day), and the rest were low-grade smokers (smoking <10 cigarettes/day). As well, most of them, 65.7%, were smoking tobacco for more than 5 years.

### Knowledge about the E-cig

**Figure 1.**
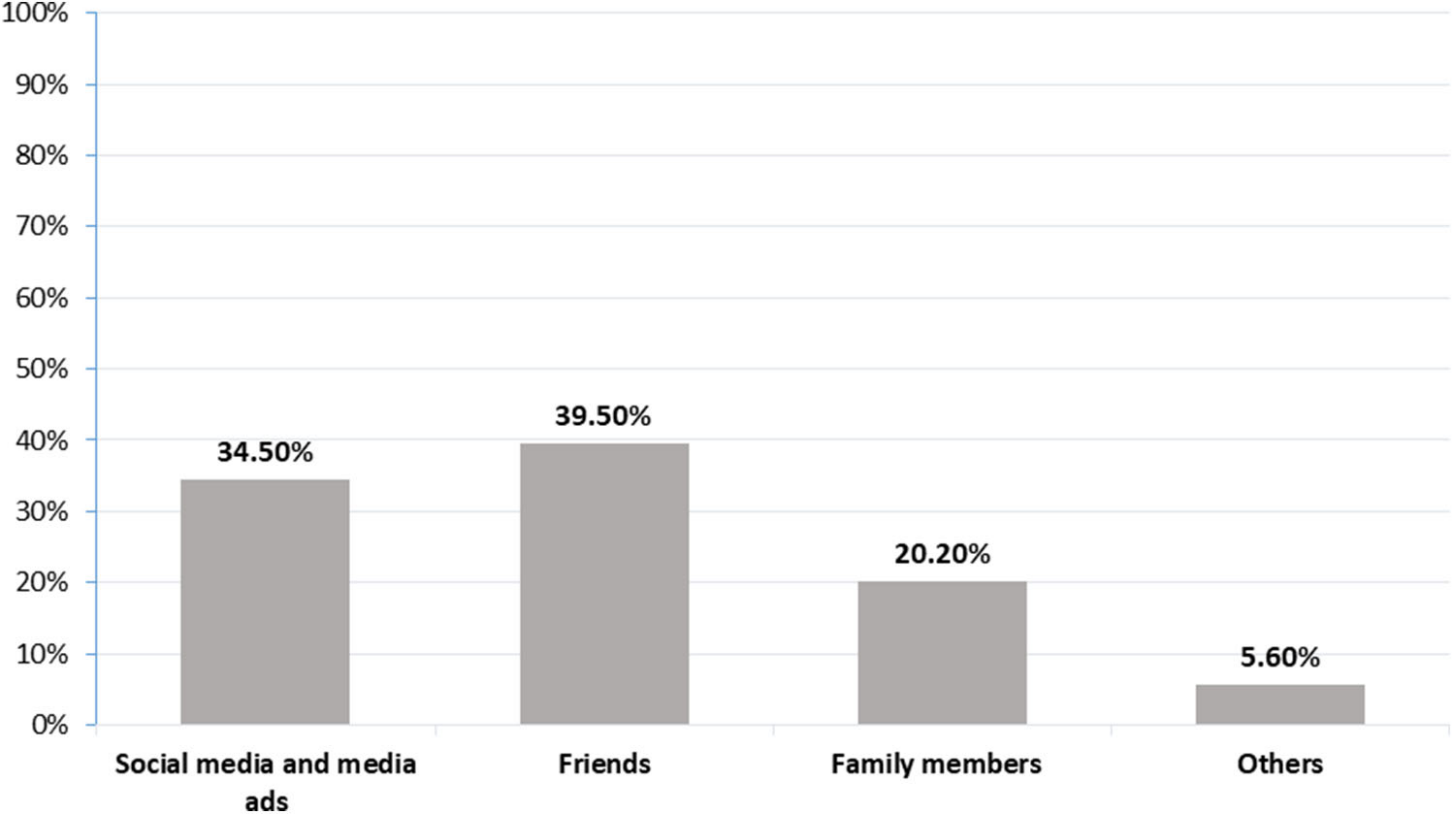
Variable source of information about the E-cigarette as reported by the study participants (*n* = 984).

**Figure 2.**
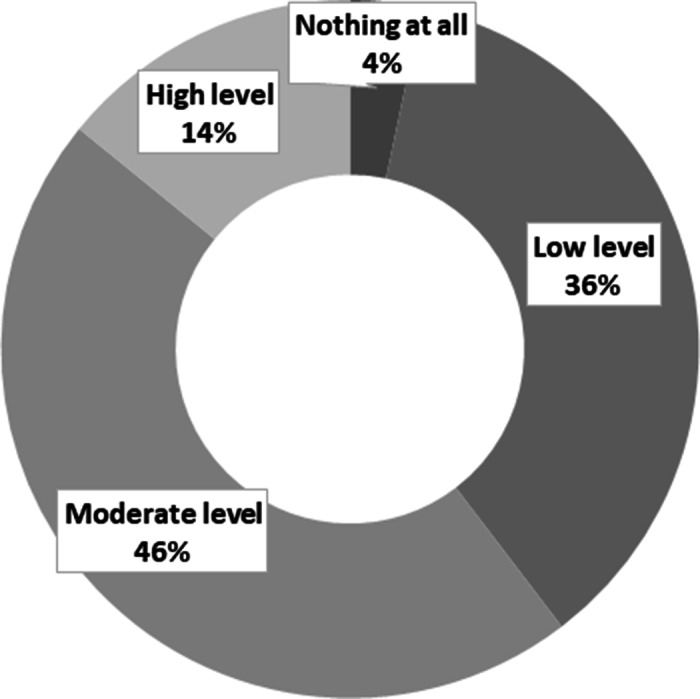
Level of knowledge as reported by the study participants about the E-cigarette (*n* = 984).

Almost all participants (*n* = 978, 99.2%) reported having heard previously about the E-cig. Many (60%) stated that the main source of their knowledge about the E-cig was from their personal contacts, including friends (*n* = 389, 40%) and family (*n* = 198, 20.2%), followed by social media and media advertisements (*n* = 339, 34.5%; Figure 1). As for the reported level of knowledge about the E-cig, only 4% of the participants did not know anything about the E-cig, while about half of them (*n* = 455, 46.2%) reported a moderate level of knowledge (Figure 2).

### Beliefs toward E-cig

In general, participants’ beliefs showed that the majority agreed that E-cig usage is a public health concern (*n* = 559, 56.8%), and it should be regulated at work and in public places (*n* = 819, 83.2%), as is the case with tobacco smoking (Table 2). Half of the study participants believed that E-cig use is cheaper and cost-effective compared to tobacco smoking (*n* = 500, 50.8%). Furthermore, around 60% (*n* = 610) of them agreed that the E-cig can be a gateway for conventional tobacco smoking, and it might lead to smoking addiction (*n* = 545, 55.4%).

**Table 2.** Participants’ reported beliefs toward the use of the E-cigarettes (*n* = 984)

	n (%)						
Questions	Nonsmokers (*n* = 492)	Tobacco smokers alone (*n* = 166)	E-cigarette users (*n* = 326)			Total n = 984	*P*-value*
			E-cig users who replaced tobacco with E-cig (*n* = 184)	Dual users E-cig+ tobacco (*n* = 52)	E-cig users without tobacco smoking history (*n* = 90)		
**Do you believe E-cigarettes are safe to use compared to tobacco cigarettes?**							0.000
○ Strongly agree/Agree	108 (11.0)	41 (4.2)	161 (16.4)	30 (3.0)	38 (3.9)	378 (38.4)	
○ Neutral	130.0 (13.2)	51.0 (5.2)	21.0 (2.1)	20.0 (2.0)	16.0 (1.6)	238.0 (24.2)	
○ Strongly disagree/Disagree	254.0 (25.8)	74.0 (7.5)	2.0 (0.2)	2.0 (0.2)	36.0 (3.7)	368.0 (37.4)	
**Do you believe E-cigarettes are safe as passive inhalation (compared with tobacco smoking)?**							0.000
○ Strongly agree/Agree	152 (15.4)	58 (5.9)	145 (14.7)	33 (3.4)	48 (4.9)	436 (44.3)	
○ Neutral	136.0 (13.8)	45.0 (4.6)	25.0 (2.5)	15.0 (1.5)	32.0 (3.3)	253.0 (25.7)	
○ Strongly disagree/Disagree	204.0 (20.7)	63.0 (6.4)	14.0 (1.4)	4.0 (0.4)	10.0 (1.0)	295.0 (30.0)	
**Do you believe the use of E-cigarettes can help in smoking cessation?**							0.000
○ Strongly agree/Agree	144 (14.6)	60 (6.1)	163 (16.6)	38 (3.9)	41 (4.2)	446 (45.3)	
○ Neutral	112.0 (11.4)	37.0 (3.8)	14.0 (1.4)	8.0 (0.8)	46.0 (4.7)	217.0 (22.7)	
○ Strongly disagree/Disagree	236.0 (24.0)	69.0 (7.0)	7.0 (0.7)	6.0 (0.6)	3.0 (0.3)	321.0 (32.0)	
**For those who switched completely from tobacco smoking to E-cigarette, do you believe that there is a positive impact on their general health status and normal life activities?**							0.000
○ Strongly agree/Agree	132 (13.4)	55 (5.6)	154 (15.7)	33 (3.4)	40 (4.1)	414 (42.1)	
○ Neutral	167.0 (17.0)	59.0 (6.0)	21.0 (2.1)	15.0 (1.5)	25.0 (2.5)	287.0 (29.2)	
○ Strongly disagree/Disagree	193.0 (19.6)	52.0 (5.3)	9.0 (0.9)	4.0 (0.4)	25.0 (2.5)	283.0 (28.8)	
**Do you think E-cigarette is cost-effective (Cheaper) compared with tobacco smoking?**							0.000
○ Strongly agree/Agree	170 (17.3)	86 (8.7)	146 (14.8)	39 (4.0)	59 (6.0)	500 (50.8)	
○ Neutral	181.0 (18.4)	42.0 (4.3)	28.0 (2.8)	10.0 (1.0)	22.0 (2.2)	283.0 (28.8)	
○ Strongly disagree/Disagree	141.0 (14.3)	38.0 (3.9)	10.0 (1.0)	3.0 (0.3)	9.0 (0.9)	201.0 (20.4)	
**Do you believe E-cigarette may be a gateway to conventional smoking?**							0.000
○ Strongly agree/Agree	342 (34.8)	110 (11.2)	81 (8.2)	26 (2.6)	51 (5.2)	610 (62.0)	
○ Neutral	92.0 (9.3)	28.0 (2.8)	44.0 (4.5)	15.0 (1.5)	17.0 (1.7)	196.0 (19.9)	
○ Strongly disagree/Disagree	58.0 (5.9)	28.0 (2.8)	59.0 (6.0)	11.0 (1.1)	22.0 (2.2)	178.0 (18.1)	
**Do you believe E-cigarette use is a Public health concern?**							0.000
○ Strongly agree/Agree	354 (36.0)	97 (9.9)	42 (4.3)	12 (1.2)	54 (5.5)	559 (56.8)	
○ Neutral	92.0 (9.3)	44.0 (4.5)	69.0 (7.0)	21.0 (2.1)	25.0 (2.5)	251.0 (25.5)	
○ Strongly disagree/Disagree	46.0 (4.7)	25.0 (2.5)	73.0 (7.4)	19.0 (1.9)	11.0 (1.1)	174.0 (17.7)	
**Do you believe E-cigarette should be regulated like other tobacco products?**							0.000
○ Strongly agree/Agree	428 (43.5)	144 (14.6)	137 (13.9)	31 (3.2)	79 (8.0)	819 (83.2)	
○ Neutral	50.0 (5.1)	18.0 (1.8)	30.0 (3.0)	12.0 (1.2)	1.0 (0.1)	111.0 (11.3)	
○ Strongly disagree/Disagree	14.0 (1.4)	4.0 (0.4)	17.0 (1.7)	9.0 (0.9)	10.0 (1.0)	54.0 (5.5)	
**Do you believe that E-cigarettes should be regulated in work and public places?**							0.000
○ Strongly agree/Agree	409 (41.6)	122 (12.4)	61 (6.2)	19 (1.9)	57 (5.8)	668 (67.9)	
○ Neutral	58.0 (5.9)	25.0 (2.5)	50.0 (5.1)	10.0 (1.0)	12.0 (1.2)	155.0 (15.8)	
○ Strongly disagree/Disagree	25.0 (2.5)	19.0 (1.9)	73.0 (7.4)	23.0 (2.3)	21.0 (2.1)	161.0 (16.4)	
**Do you believe E-cigarette can lead to smoking addiction?**							0.000
○ Strongly agree/Agree	330 (33.5)	96 (9.8)	68 (6.9)	22 (2.2)	29 (2.9)	545 (55.4)	
○ Neutral	119.0 (12.1)	48.0 (4.9)	61.0 (6.2)	16.0 (1.6)	31.0 (3.2)	275.0 (27.9)	
○ Strongly disagree/Disagree	43.0 (4.4)	22.0 (2.2)	55.0 (5.6)	14.0 (1.4)	30.0 (3.0)	164.0 (16.7)	

* Chi-square test (*P*-value<0.05)

In more detail, there were significant differences in the belief’s responses between the study subgroups (*P*-value <0.05). Noticeably, the majority of E-cig users (from all the subgroups) have a positive belief toward the safety of direct (*n* = 229, 23.3%) or passive (*n* = 226, 23%) E-cig vaping, the impact of E-cig on the general health and normal life activities in case of switching from tobacco smoking to E-cig (*n* = 227, 23.1%), and the beneficial effect of E-cig on smoking cessation (*n* = 242, 24.7%). While most of the other categories (the nonsmokers and tobacco smokers) responded either negatively or neutrally with those aspects. Moreover, among all the study subgroups, the majority acknowledged the belief on the cost-effectiveness of E-cig compared to tobacco, but it may encourage their users to smoke tobacco and leads to smoking addiction, and its use should be regulated like any other tobacco products. Surprisingly, the responses toward the belief “E-cig use is a public concern and need to be regulated at work and public areas” were varied among the E-cig users; ranged from “agree” for the users who didn’t have a smoking history to “disagree” for the users who replaced tobacco with E-cig (Table 2).

### Correlations between responses

Multivariant linear regression outcome showed a positive significant correlation (*P* < 0.01) between the usage of the E-cig, male gender, being single in the social status, and the level of participants’ reported knowledge. Besides, the results showed the participants’ use of the E-cig increased significantly (*P* < 0.05) in parallel to their positive beliefs toward its safety, the ability of the E-cig to function as a smoking cessation aid, and its cost-effectiveness (Table 3). So, those findings declared that the Jordanians’ usage of E-cig is dependent on three factors which are beliefs toward its safety, the knowledge about E-cig, and beliefs of cost-effectiveness of E-cig.

**Table 3.** Correlation between E-cigarettes usage and different variables using simple and multivariant linear regression (*n* = 984)

Variables	Linear regression		Multivariable regression	
	Beta	*P*-value	Beta	*P*-value
**Gender**, ref = male	−0.885	**<0.001**	−0.981	**<0.001**
**Social status**, ref = married				
Single	0.145	**0.001**	0.205	**0.003**
**Level of knowledge** *, ref = moderate level				
Low level	−0.374	0.202	−0.364	0.252
High level	1.885	**0.011**	1.995	**0.001**
**Believe about E-cig safety compared with tobacco, ref = neutral**				
Agree	1.224	**<0.001**	1.285	**<0.001**
Strongly Agree	1.208	**0.001**	1.442	**<0.001**
Disagree	−1.303	**0.016**	−1.344	**0.001**
Strongly Disagree	−1.235	**0.044**	−1.332	**0.047**
**Believe about the use of E-cig as a smoking cessation aid, ref = neutral**				
Agree	0.524	**0.048**	0.664	**0.040**
Strongly Agree	1.576	**<0.001**	1.895	**<0.001**
Disagree	−0.484	0.292	−0.869	0.322
Strongly Disagree	−0.625	0.406	−0.755	0.521
**Believe about the cost-effectiveness of E-cig, ref = neutral**				
Agree	0.441	**0.028**	0.422	**0.029**
Strongly Agree	0.125	**0.031**	0.225	**0.039**
Disagree	−0.662	0.197	−0.712	0.227
Strongly Disagree	−0.361	0.701	−0.895	0.821

* Level of knowledge from participants’ perception. Significance (*P* < 0.05) was presented in bold numbers.

## Discussion

Worldwide, tobacco smoking leads to more than 7 million deaths per year and more than 16 million reports for smoking-related diseases in the United States of America (USA) (CDC, 2020c). Recently, the tobacco smoking rate among Jordanians was reported as one of the highest rates in the world (Marquez *et al*., 2019). While, E-cig use is becoming more popular globally, and specifically in Jordan, as a new smoking tool, that is marketed as an alternative to conventional tobacco cigarettes. This cross-sectional study succeeded in assessing the perception of the Jordanian population toward the E-cig and evaluated their knowledge about it compared to traditional tobacco smoking. A significant prevalence of E-cig usage among Jordanians was identified in this study, with a moderate perception toward safety. Usage was shown to be affected by the level of knowledge, perceived perception of safety, and relative affordability. Positive opinion toward the use of the E-cig as an aid for tobacco smoking cessation was also identified.

Although advertising the E-cig in Jordan across the media is very limited, in this study, almost all of the participants had heard about the E-cig previously, mostly on a personal level. This was higher than any other percentage reported in the literature previously. For instance, it was found in a study conducted in Egypt in 2019 that 79.3% of the participants only were aware of the E-cig (Dwedar *et al*., August 2019). Another Egyptian study conducted in 2016 revealed that only 57.5% of the participants knew about the E-cig (Abo-Elkheir and Sobh, 2016). A study conducted in 2015 (but unpublished) (Essel, 2015) in New York City found that only 32% of the participants heard of the E-cig before study conduct. Although the E-cig was first introduced to the market in 2004, it only started to gain popularity worldwide in 2012, which justifies the above findings (Abo-Elkheir and Sobh, 2016).

In 2011, the Global Adult Tobacco Survey (Marquez *et al*., 2019) revealed that around 42% of the Jordanian population aged 15 years and above smoked tobacco. In this study, 41% of the participants revealing to be current tobacco smokers/ex-smokers (with/out E-cig use), it seems that the problem of tobacco smoking in Jordan is not declining. It is actually very high compared to the surrounding Middle Eastern countries (Marquez *et al*., 2019). The study also confirms the persistence of another fact, stated in 2011 by the Global Adult Tobacco Survey (Marquez *et al*., 2019), which is male smokers being dominant in proportion compared to females (Marquez *et al*., 2019). The Jordanian culture and higher acceptance of males’ public smoking versus females’ public smoking could be the justification for such findings (Jaghbir *et al*., 2014).

In this study, the dual E-cig users declared the use of E-cig (nicotine-containing e-liquids) interchangeably with tobacco. Such practice could be related to the situation and the place of smoking, as many of them perceived E-cig as safe, acceptable to be used in public places and it is not a public concern as tobacco (Franck *et al*., 2016). Similar findings were reported in a study conducted in Sydney, where 27% of the study E-cig users were daily tobacco smokers as well (Walsberger, 2015). It was reported in 2016, that the prevalence of dual use of E-cig with tobacco smoking in the USA was 54.6% (Truth Initiative, 2018). On the contrary, two studies conducted in Egypt (Abo-Elkheir and Sobh, 2016; Dwedar *et al*., August 2019) found that none of their study participants were dual E-cig users.

Acknowledging the source of information for health-hazardous substances is important (Keeney and Von Winterfeldt, 1986). Most of the participants in this study reported “friends” to be their main source of information on the E-cig, followed by social media and media ads. Previous studies found similar findings (Dwedar *et al*., August 2019), where about half of the participants considered media advertisement as their main source of information on the E-cig. Other studies revealed the internet, television, and friends to be different sources of information on the E-cig (Dawkins *et al*., 2013; Emery *et al*., 2014; Martinez-Sanchez *et al*., 2015). This unofficial source of information could be behind many of the misconceptions identified among the participants in this study.

The majority of the respondents in this study perceived the E-cig to be safe and cheap to use, and a good alternative to tobacco smoking. In fact, the majority of them started using it as a replacement for tobacco smoking believing that the E-cig poses lower health risks compared to tobacco smoking. Although the safety of the E-cig is still uncertain, the majority of participants reported positive beliefs regarding the safety of direct or passive vaping of E-cig compared to tobacco smoke. These results were not surprising considering previous findings unveiling perceptions and use of the E-cig among Jordanian medical students, showing similar results to this study in terms of beliefs toward the safety of the E-cig and its use (Al Oweidat *et al*., 2020). In addition, Abo-Elkheir et al conducted a study in Egypt in 2016 found that one-third of participants believed that the E-cig is less harmful compared to tobacco smoking, and about 6% reported that the E-cig is not harmful at all (Abo-Elkheir and Sobh, 2016). Such conceptions could be due to the messages delivered via the local marketing strategies showing the advantage of using the E-cig versus tobacco smoking (Cataldo *et al*., 2015). In addition, there are some shreds of evidence shows that E-cig is significantly less harmful than tobacco smoking (National Academies of Sciences, 2018) and at least as efficacious as nicotine replacement therapies for tobacco smoking cessation (Hajek *et al*., 2019). On the contrary, this conception was not the same elsewhere, as medical students in Saudi Arabia and Minnesota measured their beliefs regarding E-cig usage reported that 42% of the students did not consider the E-cig as a helpful method for tobacco smoking cessation (Hinderaker *et al*., 2018; Almutham *et al*., 2019). Of noteworthy, during August 2019, the Centers for Disease Control and Prevention reported many incidences of respiratory distress cases and injuries due to E-cig use (CDC, 2020a), questioning the real safety used in the E-cig marketing campaigns worldwide (CDC, 2020b). The CDC acknowledged that the lung problem associated with E-cig use was caused by tetrahydrocannabinol (THC) (a psychoactive substance found in the Cannabis plant) (CDC, 2020a).

In 2019, the government in Jordan imposed a 200% tax on E-cig, vapes, and paraphernalia (Marquez *et al*., 2019). In July 2020, Jordan banned tobacco smoking and E-cig usage in all indoor public places (Safi, 2020). In Jordan, E-cig is allowed legally, but the Jordan Food and Drug Association (JFDA) has published new regulations in 2019 for tobacco products and the E-cig as a starting point for its restricted usage (Jordan Food and Drug Association (JFDA), 2019). This comes in line with the latest WHO statement, urging governments to consider optimal regulation of the E-cig products, including where it is allowed to be vape (Wilson *et al*., 2017). Remarkably, more than half of the participants in this study thought that E-cig use in public places is a health concern and believed strongly that it should be regulated like other tobacco products, and should be regulated at work and public places as well. It was also reported that if vaping was allowed in public places unlike traditional cigarettes, that would encourage smokers to switch to E-cig use (Wilson *et al*., 2017). Moreover, although tobacco smoking and vaping may look similar, the uncertainty of the long-term safety of direct and passive vaping of the E-cig still stands (Wilson *et al*., 2017). With all that said, it is apparent that the government in Jordan has taken serious steps to control the use of E-cig and tobacco. Yet, holding health awareness campaigns aiming to deliver clear scientific information tailored to the different age groups of E-cig users (i.e. teenagers and adults) is still urgently needed. As well, health education programs for the E-cig handlers and vape shop workers on the emerging health issues, which could promote their knowledge and help them to answer the customers’ questions properly (Hart *et al*., 2018). To the best of our knowledge, this is the first study to assess the perception of Jordanian population about E-cig use, which could be a standpoint for the future studies which concerned about smoking behaviors and cessation aspect.

Yet, this study is not without limitations which should be taken into account when interpreting the results. These include (i) using a nonrandomized convenience sampling method, which limits the generalizability of the results. However, nowadays in light of the published review (Thornton *et al*., 2016), the recruitment of a research sample using social media platforms is considered an efficient and cost-effective method. (ii) Demographics of the Jordanian population (*n* = 10,554,000), in 2019, shows that 52.9% of the population were males, 60.3% holding bachelor university degree and 42% were living in Amman. As 26.3% of the Jordanians were aged 20–34 years old, while the ages of 35–49 and 50–65 recorded 17.7% and 8.1%, respectively (Department of Statistics, 2019). Comparing the general population demographics with the study sample, showed evidence of skewness in the sample. Descriptive analysis of the skewness statistics showed that living in Amman versus other countries was associated with high skewness (G1 = 1.7). This will limit the generalizability of study results over the Jordanian population. Further studies are required to investigate the prevalence of E-cig usage in other cities. (iii) The number of tobacco smokers participates didn’t match the high percentage of tobacco smoking rates in Jordan mentioned in (WHO, 2015). This would urge the need for further detailed studies, targeting the tobacco smokers’ perceptions (all types of tobacco, i.e. water pipes, cigarettes, etc.), believes, and perception toward E-cig. As well as the participants believe that E-cig could be a successful tool to quit tobacco smoking under strict regulations, future studies should investigate the extent of real success among users.

## Conclusion

This study illustrated a significant prevalence of E-cig usage among Jordanians with a moderate perception toward safety and showed a need for restrictive regulations. The usage level is substantially affected by the belief toward its safety, level of knowledge, and relative affordability of the E-cig among the surveyed population. This study showed a considerable level of positive opinion toward E-cig use as a helpful aid for tobacco smoking cessation. Nonetheless, there is a need for health awareness campaigns for the entire Jordanian community and healthcare workers about E-cig use, emerging related health findings, and how to promote tobacco smoking cessation.
